# *Mycoplasma synoviae* enolase is a plasminogen/fibronectin binding protein

**DOI:** 10.1186/s12917-014-0223-6

**Published:** 2014-09-25

**Authors:** Shijun Bao, Xiaoqin Guo, Shengqing Yu, Jiabo Ding, Lei Tan, Fanqin Zhang, Yingjie Sun, Xusheng Qiu, Guanghua Chen, Chan Ding

**Affiliations:** Shanghai Veterinary Research Institute, Chinese Academy of Agricultural Sciences, 518 Ziyue Road, Shanghai, 200241 China; College of Veterinary Medicine, Gansu Agricultural University, 1 Yingmencun, Lanzhou, 730070 China; China Institute of Veterinary Drug Control, 8 Zhongguancun South Street, Beijing, 100081 China

**Keywords:** *Mycoplasma synoviae*, Enolase, Enzymatic activity, Adherence

## Abstract

**Background:**

*Mycoplasma synoviae* is an avian pathogen that can lead to respiratory tract infections and arthritis in chickens and turkeys, resulting in serious economic losses to the poultry industry. Enolase reportedly plays important roles in several bacterial pathogens, but its role in *M. synoviae* has not been established. Therefore, in this study, the enolase encoding gene (*eno*) of *M. synoviae* was amplified from strain WVU1853 and expressed in *E. coli* BL21 cells. Then the enzymatic activity, immunogenicity and binding activity with chicken plasminogen (Plg) and human fibronectin (Fn) was evaluated.

**Results:**

We demonstrated that the recombinant *M. synoviae* enolase protein (rMsEno) can catalyze the conversion of 2-phosphoglycerate (2-PGA) to phosphoenolpyruvate (PEP), the Km and Vmax values of rMsEno were 1.1 × 10^−3^ M and 0.739 μmol/L/min, respectively. Western blot and immuno-electron microscopy analyses confirmed that enolase was distributed on the surface and within the cytoplasm of *M. synoviae* cells. The binding assays demonstrated that rMsEno was able to bind to chicken Plg and human Fn proteins. A complement-dependent mycoplasmacidal assay demonstrated that rabbit anti–rMsEno serum had distinct mycoplasmacidal efficacy in the presence of complement, which also confirmed that enolase was distributed on the surface of *M. synoviae*. An inhibition assay showed that the adherence of *M. synoviae* to DF-1 cells pre-treated with Plg could be effectively inhibited by treatment with rabbit anti-rMsEno serum.

**Conclusion:**

These results reveal that *M. synoviae* enolase has good catalytic activity for conversion of 2-PGA to PEP, and binding activity with chicken Plg and human Fn. Rabbit anti–rMsEno serum displayed an obvious complement-dependent mycoplasmacidal effect and adherent inhibition effect. These results suggested that the *M. synoviae* enolase plays an important role in *M. synoviae* metabolism, and could potentially impact *M. synoviae* infection and immunity.

**Electronic supplementary material:**

The online version of this article (doi:10.1186/s12917-014-0223-6) contains supplementary material, which is available to authorized users.

## Background

*Mycoplasma synoviae* is a major poultry pathogen that causes respiratory tract infection and arthritis in chickens and turkeys worldwide [1]. *M. synoviae* infections can lead to a range of diseases, from sub-clinical to severe, cause serious economic losses to the poultry industry due to retarded growth, drop in egg production, poor hatchability, downgrading at slaughter, and increased costs associated with control of the disease [2,3]. Once chickens are infected by *M. synoviae*, they become more vulnerable to other pathogens, such as Newcastle disease virus, Infectious bursal disease virus, Infectious bronchitis virus, *Escherichia coli* and *M. meleagridis*, thereby further increasing economic losses [4-9]. Relevant research on *M. synoviae* will establish a solid theoretical foundation for further development of vaccines, diagnostic reagents, and therapeutic drugs against *M. synoviae* infections.

Enolase (2-phospho-D-glycerate hydrolase) is an enzyme which catalyzes the reversible interconversion of 2-phosphoglycerate (2-PGA) and phosphoenolpyruvate (PEP). Studies have shown that enolase has many biological functions, such as its catalytic activity, binding activity with plasminogen (Plg) and fibronectin (Fn) and it can act as a heat-shock protein [10]. Aside from its enzymatic activity, the binding activity of surface-exposed enolase of microorganisms to Plg and Fn can assist pathogenic microorganisms in adhesion to host cells and dissemination within hosts, which potentially playing a role in pathogenesis [11-14]. Moreover, as a protective antigen, enolase is also an important potential candidate antigen for vaccines against infection [15-17]. *M. synoviae* enolase has also been confirmed to be a major immunogenic proteins and an extracellular protein [18-20], but most features of *M. synoviae* enolase have not been reported. Therefore, in the present study, the *M. synoviae eno* gene was cloned and expressed, and then the expression product was characterized so as to elucidate its roles in *M. synoviae* metabolism and pathogenesis.

## Results

### Cloning and expression of the *M. synoviae eno* gene

Three primer pairs (eno1F/eno1R, eno2F/eno2R, and eno3F/eno3R) were used to PCR-amplify three fragments of the *eno* gene from *M. synoviae* strain WVU 1853 and then the full length of the *eno* gene was amplified using primer pairs eno1F/eno3R. After digestion with the *Sac*I/*Xho*I restriction enzymes, the *eno* gene was cloned into the pET-28a (+) vector. Sequence analysis indicated that the *eno* gene was 1,359 bp in length and the tryptophan codon (TGA) in the gene was successfully mutated to TGG. The *eno* gene encoded a 452-amino-acid protein with a theoretical molecular weight of approximately 49 kDa, which had a 99% sequence identity with that of *M. synoviae* 53 (AE017245.1). It is also highly homologous with those of other *Mycoplasma* species (70%–80%).

**Figure 1:**
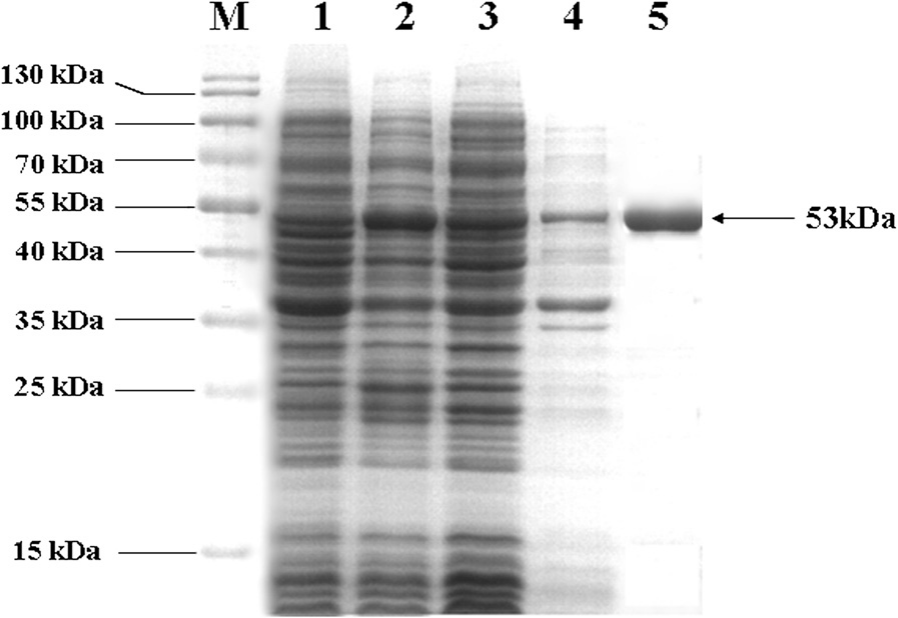
**Analysis of rMsEno expression and purification using SDS-PAGE followed by Coomassie blue staining.** M: PageRuler™ Prestained Protein Ladder (SM0671, Fermentas). Lane 1: Total cellular proteins of *E. coli* BL21 (DE3) cells transformed by pET-28a (+). Lane 2: Total cellular proteins of *E. coli* BL21 (DE3) cells transformed by pET-Eno. Lane 3: Supernatant of lysate of *E. coli* BL21 (DE3) cells transformed by pET-Eno. Lane 4: Sediment of lysate of *E. coli* BL21 (DE3) cells transformed by pET-Eno. Lane 5: Purified recombinant protein.

The recombinant pET-Eno plasmid was transformed into *E. coli* BL21 (DE3) cells. Then the recombinant rMsEno protein was expressed by induction with IPTG, assessed by SDS-PAGE with Coomassie blue staining. The results showed that more rMsEno was in the supernatant than in the sediment, and rMsEno had an apparent molecular weight of approximately 53 kDa (Figure 1, lanes 2–4). Purified rMsEno had a single band (Figure 1, lane 5).

### Measurement of the enzymatic activity of the rMsEno

**Figure 2:**
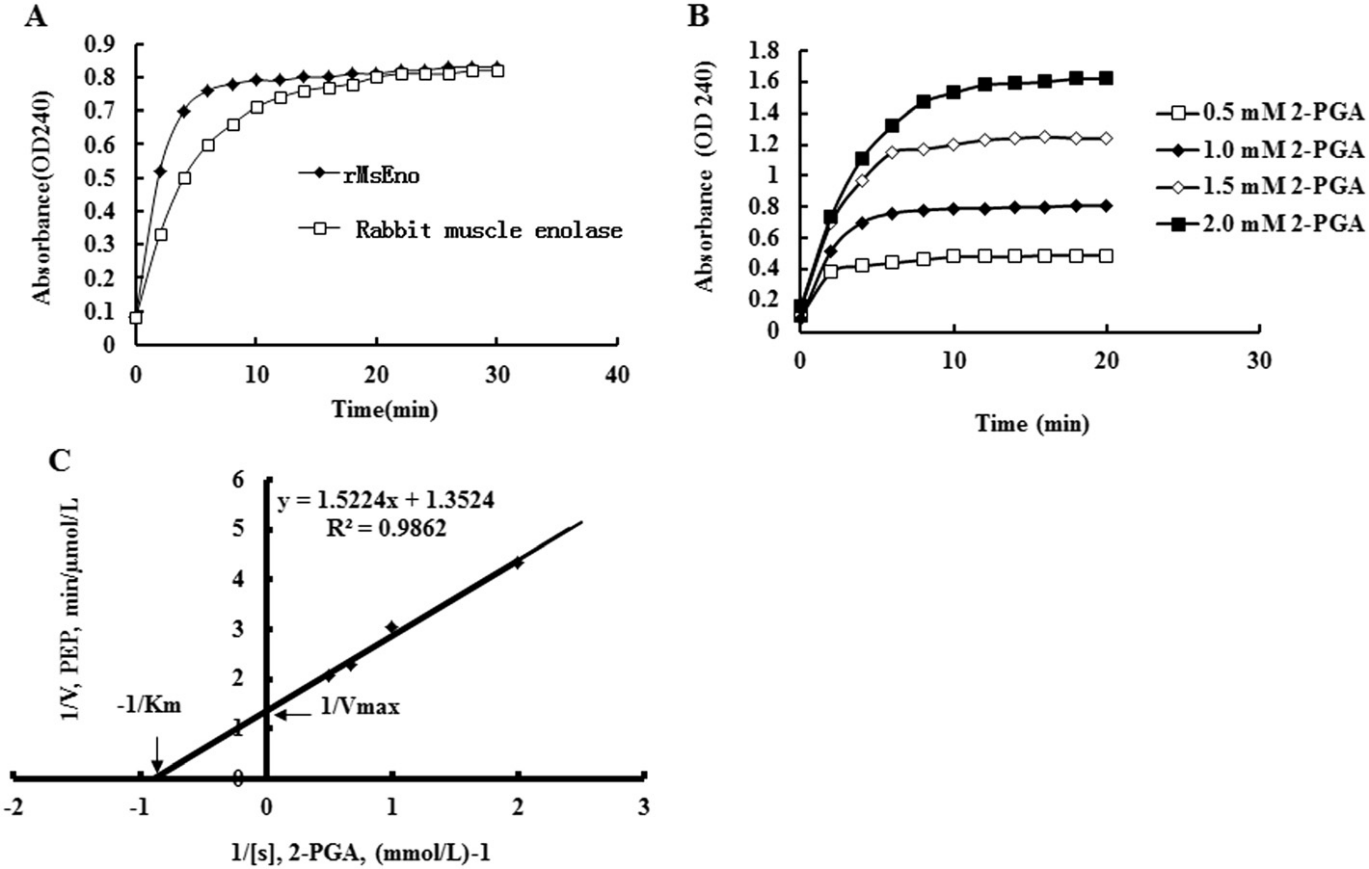
**rMsEno enzymatic activity and its influence factors. (A)** The enzymatic activity of rMsEno was determined by measuring the conversion of 2-PGA to PEP. **(B)** The effect of substrate (2-PGA) concentration on the enzymatic activity of rMsEno. **(C)** Km and Vmax for rMsEno were determined as 1.1 × 10^−3^ M and 0.793 μmol/L/min respectively, based on the Lineweaver–Burk plot (double-reciprocal plot).

The enzymatic activity of purified rMsEno was higher than that of rabbit muscle enolase in catalyzing the conversion of 2-PGA to PEP at 30°C (Figure 2A). The catalytic activity of rMsEno increased with the increase of the 2-PGA concentration from 0.5 to 2.0 mM (Figure 2B). Using the data mentioned above and applying double-reciprocal Lineweaver-Burk plots, the Km and Vmax values of rMsEno were determined to be 1.1 × 10^−3^ M and 0.739 μmol/L/min, respectively (Figure 2C).

### Subcellular localization of *M. synoviae* enolase

**Figure 3:**
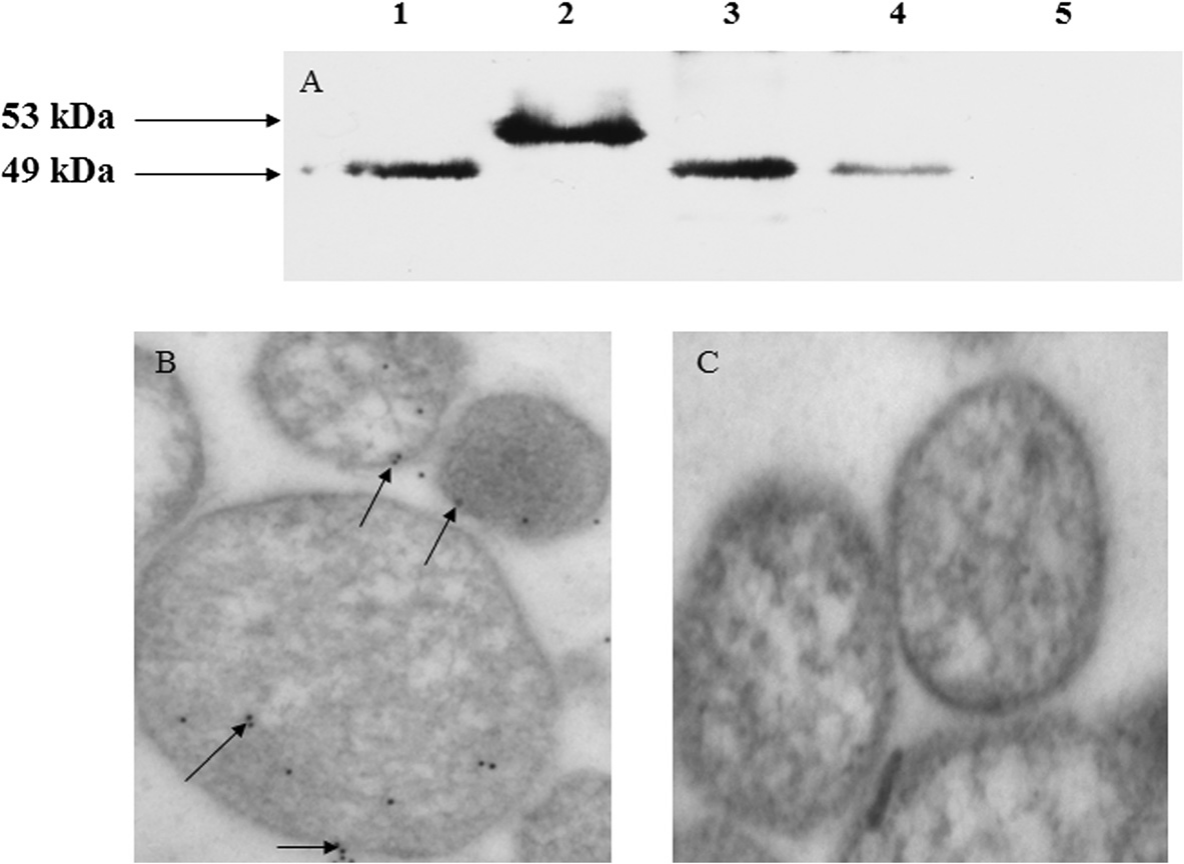
**Determination of the localization of** ***M. synoviae*** **enolase. (A)** Western blot analysis. Lane 1: Total cellular proteins of *M. synoviae*. Lane 2: Purified rMsEno was used as a positive control. Lane 3: The cytosolic proteins of *M. synoviae.* Lane 4: The membrane proteins of *M. synoviae.* Lane 5: BSA was used as a negative control. **(B)** Immunoelectron microscopy examination. Arrows pointed the *M. synoviae* enolase to be stained with goat anti-rabbit IgG labeled with 12 nm diametral colloidal gold particles. **(C)** Non-immunized rabbit serum showed no any binding activity.

Rabbit anti-rMsEno serum was isolated and the ELISA titer was determined as 1:15,000. To determine the distribution of *M. synoviae* enolase, total cellular proteins, membrane proteins and cytoplasmic proteins of *M. synoviae* were subjected to western blot analysis using rabbit anti-rMsEno serum. Purified rMsEno and BSA were used as positive and negative controls, respectively. The results showed that the rabbit anti-rMsEno serum can bind to the total cellular proteins, membrane and cytoplasmic proteins of *M. synoviae* at approximately 49 kDa (Figure 3A, lanes 1, 3, and 4). The rMsEno antiserum can also bind to purified rMsEno at approximately 53 kDa (Figure 3A, lane 2) and no specific binding strips was shown with BSA (Figure 3A, lane 5). The results also indicated that *M. synoviae* enolase existed in both membrane fractions and cytoplasmic fractions of *M. synoviae*, but the content of enolase in the cytoplasmic fractions is more than the membrane fractions. The result was further confirmed by immunoelectron microscopy with the colloid gold labeling technique (Figure 3B). Non-immunized rabbit serum showed no binding activity (Figure 3C).

### Binding activity of rMsEno to chicken Plg and human Fn

**Figure 4:**
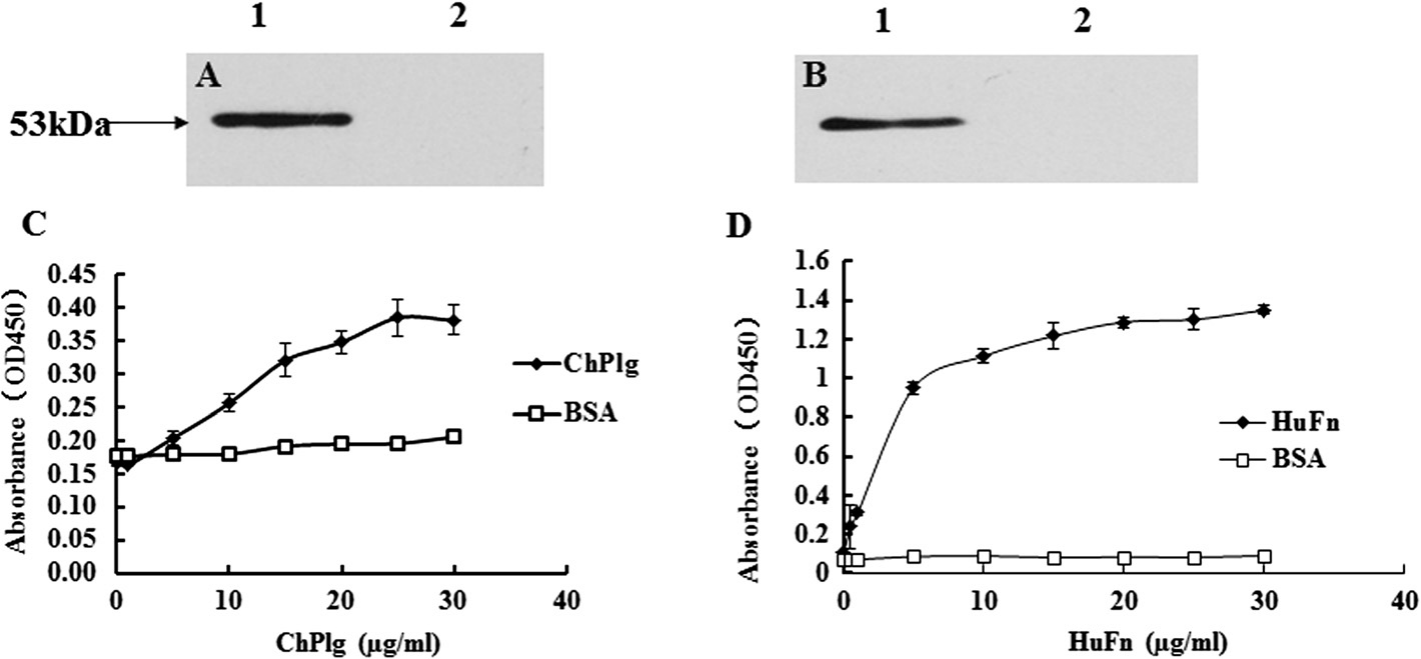
**Binding assays. (A)** Western blot analysis of the binding ability of rMsEno to chicken Plg. Lane 1: rMsEno; Lane 2: BSA. **(B)** Western blot analysis of the binding ability of rMsEno to human Fn. Lane 1: rMsEno; Lane 2: BSA. **(C)** The binding ability of rMsEno to chicken plg. **(D)** The binding ability of rMsEno to human Fn.

Western blot analysis confirmed that both chicken Plg and human Fn interacted with rMsEno with a binding band of approximately 53 kDa (Figure 4A and 4B, lane 1). Under similar conditions, BSA had no binding band (Figures 4A and 4B, lane 2). The ELISA plate binding assay showed that the rMsEno was able to bind to chicken Plg and human Fn in a dose-dependent pattern. BSA showed no interaction with either chicken Plg or human Fn (Figure 4C and 4D).

### Adherence and inhibition assays

**Figure 5:**
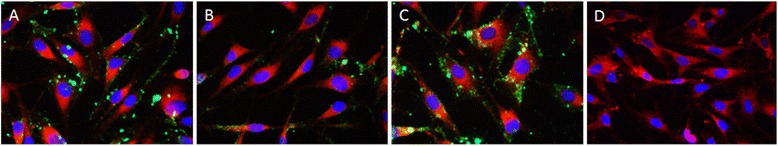
**Adherence and inhibition assays. (A)** DF-1 cells pre-treated with Plg were infected with *M. synoviae* WVU 1853 strain. **(B)** The adherence was inhibited by rabbit anti-rMsEno serum. **(C)** Treatment with non-immune rabbit serum showed no influence on the bacterial adherence. **(D)** Negative control of uninfected DF-1 cells showed no bacterial attachment when incubated with the goat anti-rabbit IgG (whole molecule)-FITC.

Adherence and inhibition assays have shown that the *M. synoviae* WVU1853 strain can adhere to DF-1 cells pre-treated with Plg (Figure 5A). And this adherence could be inhibited by rabbit anti-rMsEno serum (Figure 5B). Non-immune rabbit serum showed no effect on the adherence inhibition of *M. synoviae* to DF-1 cells (Figure 5C). Furthermore, immuno-fluorescence assay showed that no fluorescence was detected when DF-1 cells were only incubated with the goat anti-rabbit IgG (whole molecule)-FITC (Figure 5D). Therefore, all these results have demonstrated that *M. synoviae* enolase is an adhesion-related protein.

### Mycoplasmacidal assay

As shown in Table 1, compared with the rabbit pre-serum, the rabbit anti–rMsEno serum demonstrated an obvious mycoplasmacidal activity (*p* < 0.01). The mycoplasmacidal activity of the rabbit anti–rMsEno serum was similar to that of rabbit anti-*M. synoviae* serum.

**Table 1:** **Mycoplasmacidal activity of rabbit anti–rMsEno serum**

**Sera**	**Mean CFU of** ***M. synoviae*** ^**a**^	**Bactericidal coefficient (%)**
Rabbit anti-*M. synoviae* serum	13	94.09**
Rabbit anti-rMsEno serum	18	91.82**
Non-immunized rabbit serum	220	-
Blank control	280	

^a^Data were expressed as geomeans of three experiments, analyzed using the Student’s *t*-test. **p < 0.01, compared to a negative control of non-immunized rabbit serum.

## Discussion

Enolase is a multi-functional protein in both prokaryotes and eukaryotes. Aside from its enzymatic activity, enolase plays important roles in several biological and pathophysiological processes: enolase serves as a Plg receptor on the surface of a variety of hematopoietic, epithelial, and endothelial cells and plays a crucial role in intravascular and pericellular fibrinolytic systems [10,21]. Enolase has been implicated in many infectious, autoimmune, and inflammatory diseases [10,22-24]. Moreover, enolase has been identified as a protective antigen in some pathogens [15-17]. Recent studies have shown that enolase plays an important role in transcriptional regulation and can also result in immune suppression [25-27]. In the present study, the *M. synoviae eno* gene was cloned and expressed in *E. coli* cells. SDS-PAGE showed that more product of rMsEno expression were in the supernatant, which was convenient to study the catalytic activity and other biological characteristics of *M. synoviae* enolase. Subsequently, the expression products were purified and characterized. Enzymatic activity analysis showed that the purified rMsEno could catalyze the conversion of 2-PGA to PEP in reaction buffer with 10 mM MgCl_2_ at 30°C and pH 7.5.

The *M. synoviae* membrane and cytosolic proteins were isolated and analyzed to determine the subcellular localization of the *M. synoviae* enolase. By western blot and immuno-electron microscopic analyses, we demonstrated that *M. synoviae* enolase was found in the membrane and cytoplasm portions, which is consistant with the result reported previously [20], which might be more conductive to perform its different functions. *Lactobacilli* can rapidly modify their surface properties by translocation of enolase to the cell surface or releasing into the medium at different pH values [28]. Whether the *M. synoviae* enolase has similar effect will be validated in future studies. Using a complement-dependent mycoplasmacidal assay, we demonstrated that rabbit anti-rMsEno serum displayed an obvious complement-dependent mycoplasmacidal effect. It has been confirmed that *Streptococcus suis* serotype 2 enolase was a protective antigen [16]. Based on the above results, we propose that *M. synoviae* enolase may be an important surface-exposed antigen. Further studies will be designed to investigate whether *M. synoviae* enolase is a protective protein and could serve as a vaccine candidate.

The study demonstrated that *M. synoviae* enolase can bind to chicken Plg and human Fn, which is in accordance with the studies on other species of *Mycoplasma* and other microorganisms [14,29-34]. Moreover, the adherence of *M. synoviae* to DF-1 cells can be effectively inhibited by rabbit anti-rMsEno serum, which indicated that *M. synoviae* enolase is an important adhesion-related factor. That was also confirmed by adherence and inhibition assays of *M. synoviae* to the DF-1 cells pretreated with Plg. Therefore, we reasoned that enolase plays important roles in *M. synoviae* adherence, colonization, and invasion to host cells, as reported in other pathogens [14,29].

## Conclusion

In summary, the present study demonstrated that the rMsEno displayed enolase activity by catalyzing the conversion of 2-PGA to PEP. Moreover, the *M. synoviae* enolase was identified as a surface-exposed protein, and rabbit anti-rMsEno serum displayed an obvious complement-dependent mycoplasmacidal effect. In addition, the present study demonstrated that *M. synoviae* enolase could bind to chicken Plg and human Fn, and rabbit anti-rMsEno serum effectively inhibited adherence of *M. synoviae* to DF-1 cells. Therefore, we concluded that *M. synoviae* enolase plays an important role in *M. synoviae* metabolism, and could potentially impact infection and immunity. Thus further studies on the functions of *M. synoviae* enolase are warranted.

## Methods

### Enzyme and reagents

Restriction enzymes and T4 DNA ligase used in this study were obtained from MBI Fermentas (Hanover, MD, USA). PrimeSTAR® HS DNA polymerase was the product of TaKaRa (Dalian, China). Complement sera (guinea pig source) were obtained from the Control Institute of Veterinary Bioproducts and Pharmaceuticals (CIVBP, Beijing, China). The reagents used for cell culture were purchased from Gibco (Grand Island, NY, USA). All other chemicals used in this study were analytical grade and purchased from Sigma-Aldrich (St. Louis, MO, USA) or Sangon Biotech (Shanghai, China).

### Bacterial strains, cell line, plasmids and cultivation conditions

*M. synoviae* strain WVU 1853 was obtained from the Chinese Veterinary Culture Collection Center (Beijing, China) and grown in Frey’s medium [35] at 37°C in an atmosphere of 5% CO_2_. The strain was passed less than five times *in vitro. Escherichia coli* strains DH5α and BL21 (DE3) (Tiangen, Beijing, China) were cultured in Luria-Bertani (LB) broth or on solid media containing 1.2% Noble agar [36] supplemented with 50 μg/mL of kanamycin at 37°C. DF-1 cells, a continuous cell line of chicken embryo fibroblasts, were obtained from the American Type Culture Collection (Manassas, VA, USA) and certified to be free of *Mycoplasma* contamination. Cells were grown in Dulbecco’s modified Eagle’s medium (DMEM) supplemented with 10% fetal bovine serum, 100 IU/mL of penicillin, and 100 μg/mL of streptomycin at 37°C in an atmosphere of 5% CO_2_. The pET-28a (+) expression vector was obtained from Novagen (Madison, WI, USA).

### Cloning and expression of the *eno* gene

*M. synoviae* genomic DNA was extracted from strain WVU 1853 using the TIANamp Bacteria DNA Kit according to the manufacturer’s protocol (Tiangen, Beijing, China). According to the complete *M. synoviae* strain 53 genome sequence in the GenBank database (www.ncbi.nlm.nih.gov/genbank/), there are two tryptophan codons (TGA) in the coding DNA sequence (CDS) of *M. synoviae eno* gene, which are stop codons in *E. coli*. Therefore, three pairs of primers (eno1F/eno1R, eno2F/eno2R, and eno3F/eno3R; Table 2) were designed and synthesized for site-directed mutagenesis by overlap polymerase chain reaction (PCR) to amplify the *eno* gene as previously described [14]. The *M. synoviae eno* gene was amplified using the eno1F/eno3R primer pair, which introduced the *Sac*I and *Xho*I restriction enzyme sites (underlined). A full length codon-optimized *eno* gene was cloned into the pET-28a (+) vector at the *Sac*I/*Xho*I site. The amplification of the recombinant plasmid pET-Eno was performed using *E. coli* DH5α and then transformed into *E. coli* BL21 (DE3) cells. Expression of the recombinant *M. synoviae* enolase protein (rMsEno) was induced by 1 mM isopropyl β-D −1-thiogalactopyranoside (IPTG) for 4 h and purified using the Ni-NTA His-Bind® Resin kit (Novagen, San Diego, USA). The purified expression products were assessed by 10% sodium dodecyl sulfate polyacrylamide gel electrophoresis (SDS-PAGE) with Coomassie blue staining, quantified using a BCA protein assay kit (Thermo Scientific-Pierce, Rockford, IL, USA) and used directly to measure enzymatic activity or stored at −20°C for future use.

**Table 2:** **Primers used in this study**

**Primers**	**Sequence (5′ → 3′)**	**Localization (nt)** ^***c***^	**Size (bp)**
eno1F	CGA*GAGCTC* ^a^ATGTCAGCAATTAAAAAAATCC	1	202
eno1R	CACCTTTTCCACCAAA*C* ^b^CAATTAG	202	
eno2F	CTAATTG**G** ^b^TTTGGTGGAAAAGGTG	179	785
eno2R	AAATCCAGC**C** ^b^CAGTCGCTTT	963	
eno3F	AAAGCGACTG**G** ^b^GCTGGATTT	944	399
eno3R	CGG*CTCGAG* ^a^TTATTTTTTAAGATTGTA	1342	

^a^The restriction sites of GAGCTC, CTCGAG for endonuclease *Sac*I and *Xho*I were shown in italics.

^b^Nucleotide substitutions were shown in boldface.

^c^Localization was based on the nucleotide sequence of *M. synoviae* 53 *eno* gene (GeneID:3564671).

### Determination of the enzymatic activity of rMsEno

The enzymatic activity of purified rMsEno was determined by measuring the enhancement of absorbance at 240 nm (OD_240_), which reflects the conversion of 2-PGA to PEP at 30°C as previously described [37] with slight modifications. Briefly, 1 mM 2-PGA was added to pre-heated (30°C) reaction buffer (100 mM HEPES buffer, 7.7 mM KCl, 10 mM MgCl_2_, pH 7.5) followed by the addition of 10 μg purified rMsEno to initiate the reaction. The total volume of the reaction system was 2.0 mL and the reaction system was incubated at 30°C. Absorbance at OD_240_ was determined using a spectrophotometer at 1-min intervals for 30 min. Rabbit muscle enolase (Sigma-Aldrich) was used as a positive control. The enzymatic kinetics of the enolase were performed with different 2-PGA concentrations (0.5, 1.5, and 2.0 mM). According to double-reciprocal Lineweaver–Burk plots, the Michaelis constant (Km) and maximum reaction velocity (Vmax) of rMsEno were determined.

### Preparation of rabbit antisera against rMsEno and *M. synoviae* whole cells

Rabbits were purchased from Shanghai Jia Gan Bio-Technique Co. Ltd. (Shanghai, China) and housed in cages with access to water and food *ad libitum* under biosafety conditions. Animal experiments were carried out in accordance with the Institutional Animal Care and Use Committee (IACUC) guidelines set by Shanghai Veterinary Research Institute, the Chinese Academy of Agricultural Sciences (CAAS) and checked as Additional file 1. This animal study protocol (12–02) was approved by the IACUC of the Shanghai Veterinary Research Institute, CAAS, China. To prepare polyclonal antibodies against *M. synoviae* enolase and *M. synoviae* whole cells, **t**wo New Zealand white rabbits (female; body weight, 2–3 kg) were immunized four times at 2-week intervals by subcutaneous injection with purified rMsEno protein (800 μg) or *M. synoviae* inactivated whole cells (10^10^ CFU, inactivated with 0.4% formalin at 37°C for 16 h), mixed with Imject® Alum adjuvant (Thermo Scientific-Pierce, Waltham, MA, USA), respectively. Seven days after the fourth immunization, blood samples were obtained from the rabbits and centrifuged to obtain serum. The antibody titers were measured by indirect ELISA, which the 96-well ELISA plate was coated with 100 μL coating buffer (1.5 g/L Na_2_CO_3_, 2.93 g/L NaHCO_3_, pH 9.6) containing 10 μg/mL rMsEno or *M. synoviae* whole cells (10^7^ CFU/well). Then aliquots of the serum samples were placed in 1.5-mL Eppendorf tubes and stored at −40°C for future use.

### Subcellular localization of *M. synoviae* enolase

To determine the distribution of enolase in *M. synoviae*, membrane proteins and cytoplasmic proteins of *M. synoviae* were extracted using the ReadyPrep™ Protein Extraction Kit (Membrane I) (Bio-Rad) according to the manufacturer’s instructions, then equal volumes of membrane proteins and cytoplasmic proteins were used for western blot as follows: each protein sample were loaded into each lane of an 10% polyacrylamide gel and subjected to SDS-PAGE (80 V, 180 min). Subsequently, the proteins were transferred to nitrocellulose membranes (Whatman GmbH, Staufen, Germany) at 250 mA for 90 min. Then the membrane was blocked with 5% skim milk for 3 h at room temperature. After washed three times with PBST (PBS containing 0.05% Tween-20), the membrane was incubated with rabbit anti-rMsEno serum (dilution, 1:3,000) at 4°C overnight. The membrane was washed three times and then incubated with goat anti-rabbit immunoglobulin G (IgG) conjugated to horseradish peroxidase (HRP; Sigma-Aldrich; dilution, 1:8,000) at room temperature for 1.5 h. After three times washing, the membrane was stained with enhanced chemiluminescence (ECL) reagent. Reactions were detected by exposure on an X-ray film. The total protein of *M. synoviae* was prepared by bacterial lysis buffer (Sangon, Shanghai, China) according to the manufacturer’s instructions and western blot analysis was performed as described above. Purified rMsEno (1.5 μg) was used as a positive control and BSA (1.5 μg) as a negative control.

To further validate the distribution of enolase within the cells, *M. synoviae* were grown to the mid-logarithmic phase and centrifuged at 5,000 × g for 10 min at 4°C. After washing three times using PBS (0.1 M, pH 7.2), the samples were fixed with 4% paraformaldehyde and 0.5% glutaraldehyde at room temperature for 1–2 h and rinsed three times in PBS for 15 min each. After centrifugation, the pellets were dehydrated with 70% ethanol twice for 30 min each, then 80% and 95% ethanol once for 15 min each. Cells were collected by concentration and infiltrated with 95% Ethanol: LR White (1:2) for 1 h, then 100% LR White (Sigma-Aldrich) twice for 1 h and overnight respectively. Embedment were performed with LR White by exposure to UV light (365 nm) at −20°C. After blocked with 0.05 M glycine solution for 10 min and 2% BSA for 1 h, the cells were incubated with rabbit anti-rMsEno serum (1:1,000) at 4°C overnight. Each sample was rinsed six times with a fixative for 10 min each times and re-suspended in a 1:100 dilution of goat anti-rabbit IgG labeled with 10 nm colloidal gold particles (Sigma-Aldrich). After 2 h incubation at room temperature, the cells were rinsed five times with PBS at 5-min intervals, then the samples were fixed with 2% glutaraldehyde at room temperature for 10 min, rinsed with water, colored with 4.0% uranyl acetate for 15 min, rinsed with water again, dried, and then observed by transmission electron microscopy. Non-immunized rabbit serum acts as a negative control.

### Binding activity of rMsEno to chicken Plg and human Fn

Western blot and ELISA plate binding assay were used to determine the binding activity of rMsEno to chicken Plg (Cell Sciences) and human Fn (Sigma-Aldrich). For western blot analysis, 10 μg of purified rMsEno was loaded into each lane on the 10% polyacrylamide gel and electrophoresed. Bovine serum albumin (BSA; Sigma-Aldrich) (10 μg) was used as a negative control. The proteins were transferred to membranes, which were blocked with 5% skim milk in PBST at room temperature for 3 h as described above, then washed three times with PBST, and incubated with 10 μg/mL of chicken Plg or human Fn in PBST at 37°C for 2 h. After washing three times with PBST, the membrane was incubated with rabbit anti-chicken Plg monoclonal antibody (Cell Sciences; dilution, 1:3,000) or mouse anti-human Fn monoclonal antibody (Sigma-Aldrich; dilution, 1:1,000) in PBST at room temperature for 1.5 h, respectively. After washing, the membrane was incubated with goat anti-rabbit IgG-HRP (Sigma-Aldrich; dilution, 1:8,000) or goat anti-mouse IgG-HRP (Sigma-Aldrich; dilution, 1:8,000) at room temperature for 1.5 h. After washing three times with PBST, the membrane was colored using an ECL kit (Amersham Pharmacia Biotech, Piscataway, NJ, USA) according to the manufacturer’s instructions. Reactions were detected by exposure on an X-ray film.

For the ELISA plate binding assay, the 96-well plates were coated with purified rMsEno at 10 μg/well and incubated at 4°C overnight. After being washed three times with PBST, the wells were blocked with 5% skim milk in PBST at 37°C for 2 h. The plates were three-times washed again, and added with chicken Plg or human Fn (0, 1, 5, 10, 15, 20, 25, or 30 μg/mL in PBST) for a 2 h-incubation at 37°C. After washing, the plates were incubated with rabbit anti-chicken Plg IgG fraction pAb (100 μL per well; Cell Sciences; dilution, 1:3,000) or mouse anti-human Fn monoclonal antibody (100 μL per well; Sigma-Aldrich; dilution, 1:3,000) at 37°C for 1.5 h. After washing, goat anti-rabbit IgG-HRP or goat anti-mouse rabbit IgG-HRP were added to wells (100 μL per well; Sigma-Aldrich; dilution, 1:5,000) and the plates were incubated at 37°C for 1 h. Finally, the color reaction was performed by adding soluble TMB substrate solution (TIANGEN, Beijing, China) at 100 μL per well and incubated for 10 min at room temperature. The reaction was stopped with 2 M H_2_SO_4_. Absorbance at OD_450_ was measured using a spectrophotometer. Wells coated with BSA (10 μg/well) were used as negative controls.

### Adherence and inhibition assays

To validate the adherence function of *M. synoviae* enolase to DF-1 cells, the monolayer DF-1 cells in a 35-mm dish were washed three times with DMEM and incubated with Plg (5 μg/mL) at 37°C in 5% CO_2_ for 2 h. After washing, the cells were infected with *M. synoviae* WVU 1853 strain at 200 multiplicity of infection (MOI) for 2 h. The infected DF-1 cells were washed with PBS to remove non-adherent *Mycoplasma*, and fixed with 4% paraformaldehyde (PFA). The fixed cells were washed with PBST and incubated with rabbit anti-*M. synoviae* serum (1:1,000) at 37°C for 2 h. After three washes with PBST, the cells were incubated with goat anti-rabbit IgG (whole molecule)-FITC (Sigma-Aldrich; dilution, 1:500) at 37°C for 1.5 h. The cell membranes were labeled with 500 μL of 10 μM 1,1′-dioctadecyl-3,3,3′,3′-tetramethylindocarbocyanine perchlorate (Beyotime, Jiangsu, China) at room temperature for 10 min. After five washes with PBST, the cell nuclei were labeled with 500 μL of 0.1 μg/mL of 4′, 6-diamidino-2-phenylindole (Beyotime) at room temperature for 10 min. Lastly, the cells were mounted and observed using fluorescent microscopy (Ti-S; Nikon, Tokyo, Japan) to evaluate the adherence. For the adherence inhibition assay, the *M. synoviae* was incubated with rabbit anti-rMsEno serum (dilution, 1:5) at 37°C for 30 min. After centrifugation at 10, 000 × g for 15 min, the *M. synoviae* cell pellets were re-suspended and added to the DF-1 cells to assess adherence as described above. All experiments were performed in triplicate and repeated three times.

### Complement dependent mycoplasmacidal assay

To determine the complement-dependent mycoplasmacidal activity of rabbit anti–rMsEno serum, *M. synoviae* were grown to mid-logarithmic phase, washed three times with PBS by centrifugation at 5,000 × g for 10 min at 4°C, and re-suspended in PBS at the final concentration of 6 × 10^3^ CFU/mL. The reaction system was established as follows: 160 μL of *M. synoviae* suspension and 60 μL of rabbit anti-rMsEno serum (1:5) were gently mixed in 1.5-mL microcentrifuge tubes and incubated at 37°C for 30 min. Then, 30 μL of diluted complement (1:10) or PBS were added, mixed, and incubated at 37°C for 1 h. The reaction mixture (50 μL) was spread onto solid media in a 60-mm dish and incubated at 37°C in 5% CO_2_ for 7 days to count the colonies. Rabbit anti-*M. synoviae* serum and non-immunized rabbit serum were employed as positive and negative controls, respectively. In addition, controls for complement and PBS were included. All sera except for complement used in the experiment were inactivated at 56°C for 30 min. Three independent experiments were performed in triplicate. The mycoplasmacidal coefficient was calculated as follow: [(CFU of pre-serum treatment-CFU of antiserum treatment)/(CFU of pre-serum treatment)] × 100.

## Additional file

Additional file 1:
**Completed ARRIVE checklist.**

## Abbreviations

Plg: Plasminogen
Fn: Fibronectin
2-PGA: 2-phosphoglycerate
PEP: Phosphoenolpyruvate
LB: Luria-bertani
DMEM: Dulbecco’s modified eagle’s medium
CDS: Coding DNA sequence
PCR: Polymerase chain reaction
IPTG: β-D −1-thiogalactopyranoside
SDS-PAGE: Sodium dodecyl sulfate polyacrylamide gel electrophoresis
IgG: Immunoglobulin G
ECL: Enhanced chemiluminescence
HRP: Horseradish peroxidase
TMB: 3,3,5,5-tetramethylbenzidine
CFU: Colony-forming units
rpm: Revolutions per minute
DNA: Deoxyribonucleic acid
